# Clinical Results and MRI Evaluation of Patellar Osteochondral Fracture Fixation following Patellar Dislocation

**DOI:** 10.1155/2019/7943636

**Published:** 2019-12-17

**Authors:** Krzysztof Małecki, Kornelia Pruchnik–Witosławska, Dominika Gwizdała, Piotr Grzelak, Paweł Flont, Kryspin Ryszard Niedzielski

**Affiliations:** ^1^Clinic of Orthopaedic and Traumatology, Polish Mother's Memorial Hospital Research Institute, 93-338 Łódź, ul. Rzgowska 281/289, Poland; ^2^Radiology Department, Polish Mother's Memorial Hospital Research Institute, 93-338 Łódź, ul. Rzgowska 281/289, Poland

## Abstract

**Aim:**

The aim of the study was to analyze the clinical results and MRI scans after transpatellar osteochondral fracture fixation following patellar dislocation.

**Methods:**

Our study group comprised 17 patients with patellar dislocation followed by osteochondral fracture of the articular surface of the patella. All patients underwent surgery where the fractured osteochondral fragments of the patella were attached using the transpatellar suture technique. The mean age at the time of surgery was 14.1 years, and the mean follow-up period was 7.5 years.

**Results:**

The results of the patellar compression test and the apprehension test were negative in all patients. The mean Lysholm and Kujala scores were 89.2 and 89.6, respectively. The MRI scan revealed healing of the fixed fragment and restoration of the articular surface in all patients. In 16 cases, subchondral bone of the fixed fragment area was described as irregular: its articular cartilage was narrowed and not homogenous. Progressive degenerative changes were observed in the patellofemoral joint at follow-up in three patients.

**Conclusions:**

By fixing osteochondral fragments, the patellar articular surface can be restored. The MRI scans show that the cartilage in the reconstructed surface is narrowed after a mean 7.5-year follow-up.

## 1. Introduction

Osteochondral fracture (OCF) of the patella or lateral femoral condyle is rarely described in adults but occurs more frequently in adolescents during the first or, more rarely, recurrent patellar dislocation [1–4]. Due to the risk of complications manifested by early degenerative lesions in the patellofemoral joint, early diagnosis and operative treatment are required. OCFs can be highly suspected in knees with intra-articular hematoma following patellar dislocation. Due to the difficulty in diagnosis, including even the failure to diagnose OCF, many studies highlight the need for multimodal diagnostics, including radiography (X-ray), computer-assisted tomography (CAT), or magnetic resonance imaging (MRI) [2, 3].

Although fast and accurate diagnosis increases the possibility of restoring the articular surface by fixing the fractured fragment, no studies have so far reported the long-time clinical results including a MRI scan of the knee. Available articles are primarily based on case reports and low-number case series [2, 5–11]. Only a few articles are based on large case series with clinical and MRI follow-up [3, 12]. Depending on the fragment size and fractured subchondral bone thickness, different surgical procedures are applied to treat OCF: fixation with headless metal screws or bioabsorbable pins or suture [11–14]. If the subchondral bone is too small, it is necessary to remove a fractured fragment, apply the microfracture technique, or make use of some other means of chondral plasty [4].

The aim of the study was to analyze the clinical results and MRI scans after operative fixation of OCF fragments of the patella following patellar dislocation.

## 2. Material and Methods

### 2.1. Study Group

Our study group comprised 17 patients (7 girls and 10 boys) with osteochondral fracture of the articular surface of the patella following patellar dislocation. All patients underwent surgery where fractured osteochondral fragments of the patella were attached using the transpatellar suture technique. All osteochondral fragments were from the weight-bearing area of the patella and were larger than 1 cm^2^. The cohort consisted of patients with the mean age of 14.1 years (range 13–17) and the mean follow-up period of 7.5 years (range 3–10). Preoperative diagnosis of the articular surface fracture was based on X-ray imaging: a shadow of the fractured osteochondral fragment was observed in each patient. CT or MRI examinations were performed in some cases to confirm the diagnosis and exclude the probability of other lesions. There were no top athletes among the patients.

### 2.2. Inclusion Criteria

- Diagnosis of osteochondral fracture of the patella after acute patellar dislocation
- Age under 18 years
- Acute, first time dislocation
- Follow-up time >3 years
- Operative fixation of patellar osteochondral fragment

### 2.3. Exclusion Criteria

- Over one month from injury
- Recurrent dislocations
- Fragment not suitable for fixation
- Incomplete documentation

### 2.4. Surgery Technique and Postoperative Treatment

**Figure 1:**
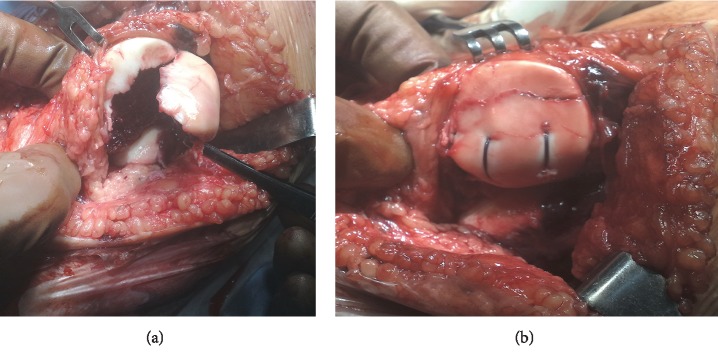
(a) Massive osteochondral fracture of the patella before fixation. (b) The same fracture after PDS transpatellar suture fixation.

Lateral or medial arthrotomy was performed depending on the preference of the surgeon and the lesion pattern. The articular surfaces were inspected to assess a magnitude of damages. After finding a loose fragment, the thickness of the osseous layer was measured and the technical possibility of its fixation was assessed. A lodge was prepared in the patella after debridement and applying the microfracture technique. The fragments were attached using a double or single transpatellar suture (PDS No. 0 sutures). Consequently, a homogenous chondral surface was reconstructed and the fragment was well stabilized (Figures 1(a) and 1(b)). Additionally, if necessary, the torn medial patellofemoral ligament and medial retinacula were sutured and overlapped. In cases of suspected ligamentous or meniscal lesions (three cases) or those with an unclear fracture site (five cases), diagnostic arthroscopy was performed before open surgery.

Over the postoperative period, the patient was immobilized for a six-week period in an orthosis or cylinder cast with no weight bearing. After cast/orthosis removal, an ambulatory rehabilitation program was initialized with quadriceps training and range of motion restoration. After six weeks, weight bearing was allowed with a gradual increase to full weight after four weeks following cast/orthosis removal; from this point, maximal quadriceps load was allowed exclusively. No CPM machine was routinely used. The presence of a full range of knee motion and normal quadriceps strength completed the rehabilitation program. The patients were allowed to return to sport activities six months postsurgery.

### 2.5. Clinical Assessment

All subjects were asked to fill in the Lysholm (Tegner modification) and Kujala scoring questionnaires and underwent a clinical examination including the apprehension test, patellar compression test, and assessment of a range of the knee motion [15, 16].

### 2.6. MRI Assessment

Examinations were performed using the Achieva 3.0 T device and a dedicated tube in the following sequences: T1W, T2W, 3D WATS, PDW, and SPAIR. Scans were evaluated separately by two radiologists. At least three years after surgery (7.5 years on average: range 3–10 years), a checkup MRI was performed to assess four aspects of the healing process of the osteochondral fragment: outline of the damaged subchondral layer compared to the intact surface, cartilage thickness in the fragment compared to the surrounding articular cartilage and its signal, and possible secondary degenerative lesions.

All procedures performed involving human participants were in accordance with the ethical standards of the institutional research committee, as well as with the 1964 Helsinki declaration and its later amendments or comparable ethical standards. Consent was obtained from the institutional bioethics commission to perform the study, and informed consent was obtained from each patient before taking part. The data supporting the findings of this study are available from the corresponding author (KM), upon reasonable request.

## 3. Results

**Figure 2:**
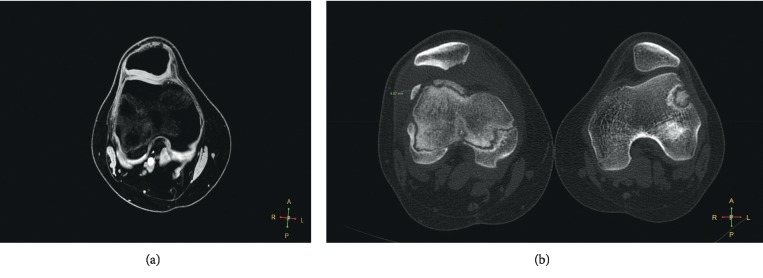
(a) Very good result with perfect OCF healing in follow-up examination in MRI 3D WATS. (b) CT scan preoperatively.

**Figure 3:**
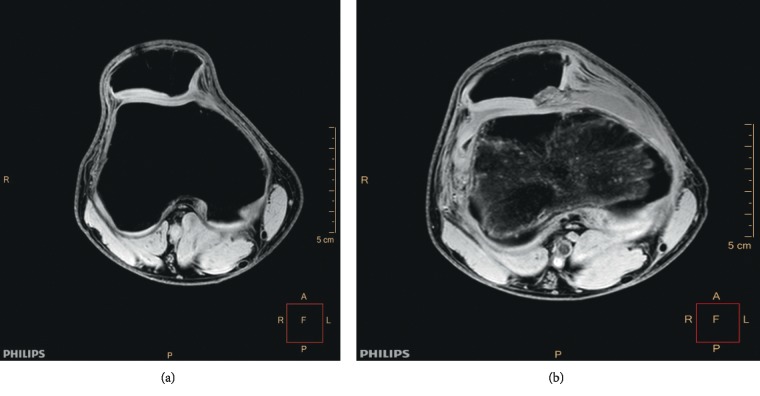
(a) Restored articular surface with irregular chondral healing in follow-up MRI WATS 3. (b) Preoperative MRI WATS 3D in the same patient.

**Figure 4:**
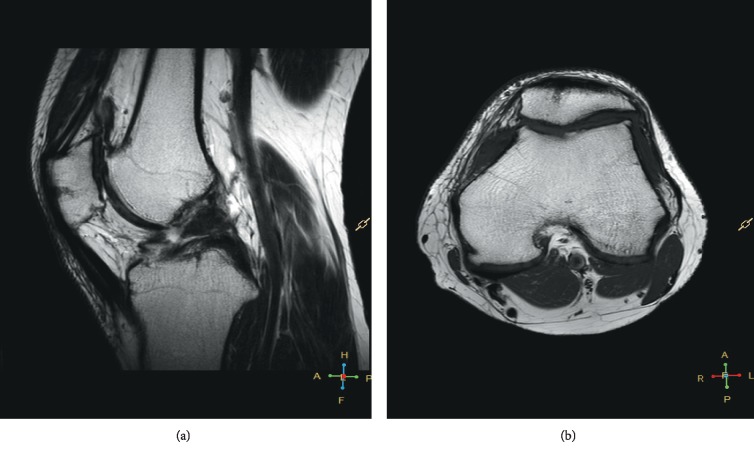
(a) Follow-up MRI T2 sagittal—osteophytes in the patellofemoral joint in one of the three patients with degenerative changes. (b) Follow-up MRI T1 axial in the same patient.

Clinical examination revealed significant limitation of the range of motion in one patient (0–90 degrees), while full functional range of knee motion was observed in all other patients. The results of the patellar compression test and the apprehension test were negative in all patients. The average Lysholm and Kujala score was 89.2 (SD 5.7) and 89.6 (SD 4.9), respectively. The MRI scan revealed healing of the fixed fragment and restoration of the continuity of the articular surface in all patients; however, while a regular outline of the subchondral bone, full thickness, and regular outline of the cartilage were observed in one female patient (Figures 2(a) and 2(b)), the subchondral bone was described as irregular and the articular cartilage was narrowed and not homogenous in the remaining 16 cases (Figures 3(a) and 3(b)). A cartilage signal of the implanted fragment corresponded to the signal of the surrounding hyaline cartilage in all cases. In three patients, progressive degenerative changes in the patellofemoral joint were observed at the follow-up examination (Figures 4(a) and 4(b)). None of the patients reported a recurrent patellar dislocation during the follow-up period.

## 4. Discussion

This study shows that the long-term outcome of fragment fixation of the patella after patellar dislocation is good. However, the cartilage in the fixed fragment is not full featured with the subchondral bone possessing a narrowed, irregular outline (Figures 3(a) and 3(b)). The excellent MRI finding observed in one female patient probably resulted from the larger thickness of the subchondral bone layer in the fixed fragment: the only case where the subchondral bone was over 4 mm thick (Figures 2(a) and 2(b)). The follow-up period in this female patient was four years; a checkup revealed a full range of knee motion, with Lysholm and Kujala scores of 95 and 94, respectively.

In three patients, MRI scan revealed initial degenerative lesions in the patellofemoral joint and, to a lesser extent, the tibiofemoral joint (Figures 4(a) and 4(b)). The examination findings may suggest that despite correct healing of the fracture, healed articular cartilage may trigger degenerative lesions over a longer follow-up if it is not at full thickness, and this will require further observation. In our opinion, as degenerative changes may be also caused by other potential constitutional factors, such as patellar maltracking, muscular disbalance, and dysplastic patellofemoral joint, it is difficult to assess the impact of surgery on the development of degenerative changes. One patient showed a limited range of motion (up to 90 degrees) most probably due to the process of rehabilitation being incomplete. Interestingly, in the study group including only patients with first time patellar dislocation, none showed recurrent patellar dislocation over the follow-up period, most probably due to the repair of the medial retinaculum and MPFL, as well as regular rehabilitation. Patients with recurrent dislocation may have a worse clinical outcome and suffer from early degenerative changes more frequently.

Whenever intra-articular hematoma is diagnosed, apart from ligament and meniscus injuries, it is most likely caused by patellar dislocation accompanied by osteochondral fracture. In such cases, X-ray examination including Merchant's view should be performed and, in the case of any doubts, an MRI test should be performed. Interestingly, detailed analysis of the anteroposterior, lateral, and axial X-ray projections performed throughout our study revealed a shadow of the fractured fragment, which suggested either a need for more extensive diagnostics or surgical intervention. This observation is not in line with previous findings, indicating that only 36% of OCFs were diagnosed in the X-ray examination [17].

Although we could not find any identical studies from the literature, some similar studies have been performed. Li et al. assessed the results of transpatellar suture in 18 patellar OCF cases with a three-year follow-up, the results being based on Lysholm and Kujala scales and MRI imaging. However, this article differs from the present study in that it includes recurrent dislocators as well as adults. No redislocations were observed and good clinical outcomes were presented [18]. Pritsch et al. described six cases of transpatellar suture OCF fixation with no complications in a two-year follow-up; however, neither functional scores nor MRI were used [19]. Lee et al. reported nine cases involving fixation and nonfixation of the osteochondral fragment using headless screws or bioabsorbable pins in a study with a 30-month follow-up. The final outcome was evaluated by the clinical IKDC and KOOS scales, and MRI was not performed [2]. However, Ghiokas et al. reported similar outcomes to the present study, i.e., they report good consolidation of the subchondral bone and partially reduced thickness of the cartilage in the location of fixation. The study included 18 adolescent OCF cases, including seven patellar cases treated with ORIF, where bioabsorbable pins were applied. The final outcomes were evaluated by MRI after 6-, 12-, 24-, and 36-month postoperative periods [12]. Sinikumpu and Serlo applied a similar operative procedure in one case and achieved a good early outcome [9].

Lidder et al. presented a case series of nine patients with osteochondral fractures, where the surgical procedure involved compressing screw fixation; the outcomes were evaluated using the KOOS, IKDC, and Lysholm scale. The average score was 96 according to the Lysholm scale and the average follow-up was 26 months [8]. A successful case of patellar OCF screw fixation was also described by Albuquerque et al. [7]. This is a good and widely recognized method of OCF fixation but, as noted by Aydogmus et al., one that also involves a risk of possible implant penetration into the joint [6]. A multicenter study by Chotel et al., reported satisfactory results from the treatment of 14 patients with OCF, including nine patients with patellar OCF; the average follow-up period was 30 months and clinical outcomes were evaluated according to the IKDC rating scale [1]. Dhawan and Hospodar discussed a single-case report on the outcome of the operative suture fixation of the patellar OCF. They reported good results according to MRI scans performed nine weeks after surgery [11]. In contrast, Seeley et al. described a group of 46 adolescents presenting with acute patellar dislocation with some form of displaced OCF, 35 of whom with patellar OCF. Operative treatment was performed in 20 cases: ORIF was applied in only two cases, while a loose body was removed or other chondral restoration procedures were performed in the others. No follow-up MRI scans were performed. No significant differences in IKDC scores were observed between loose body removal and ORIF at one-year follow-up; however, any results obtained on the basis of such a small group of ORIF patients should be interpreted cautiously [3].

Given the research techniques, homogeneous study group and quite a long follow-up period, our study allows for a comprehensive evaluation and conclusions. The disadvantage of this study is that it is retrospective in its nature; furthermore, we are reporting on only one fixation method. Comapring another ORIF method of fixation would have added the value of this study.

Many different methods of fixation are described in the literature, including suture, bioabsorbable pins, and Herbert screw; transpatellar PDS suture proved to be effective and inexpensive in osteochondral fractures greater than 1 cm^2^ within the weight-bearing area [11, 18, 19]. The suture method is suitable for young patients and is not associated with the risk of implant penetration to the joint and secondary need of its removal [6, 11]. However, one disadvantage of the technique is that it offers questionable stability when used with large fragments; nevertheless, this problem can be solved through the precise placement of a sufficient number of sutures [13, 18]. Although Herbert or bioabsorable screws may provide more compression than sutures, this approach becomes more challenging for smaller fragments. Nevertheless, in our opinion, transpatellar suture offers great promise in the treatment of patellar OCF.

The main disadvantage of the open surgery is that it may leave a significant scar; however, due to the technical difficulties and limitations associated with arthroscopic surgery, it was not performed for suture fixation in the present study.

Fixation of the osteochondral fragment allows for the reconstruction of the patellar articular surface. The MRI scans show that the cartilage in the reconstructed surface is narrowed after an average of 7.5-year follow-up. It should be emphasized that the diagnosis of the osteochondral fracture following patellar dislocation is crucial since failed diagnosis is known to result in accelerated degenerative changes.

## Data Availability

The data supporting the findings of this study are available from the corresponding author upon reasonable request.
